# Absolutely Pure Gold for Filling Teeth

**Published:** 1867-06

**Authors:** G. S. Mills


### ABSOLUTELY PURE GOLD FOR FILLING TEETH.

BY G. S. MILLS, D.D.S.

THE Dental profession will bear me out in the assertion, that for the past two years, there has been more said and done to produce a superior quality of gold, for filling teeth, than ever before. Competition is said to be the life of trade. Rivalry for good is commendable in all. Whatever motives have prompted the manufacturers, I leave that aside. Suffice it, we have to-day, a better article in the market by the undertaking. Great effort has been made to suit all parties, and for this reason the beaters of gold have failed to secure the best results. Some desire foil to be very adhesive, others cannot use it if it is; another wishes it entirely non-adhesive, trusting to the welding process to retain the filling, "which I believe to be, in these days of progress, a very old fogy idea;" nevertheless, it is a free country, and I know of no law that prevents a man from spending double the amount of time to accomplish a good result, that is necessary. It is not good for man to be alone, (hence a good mallet, and assistant,) notwithstanding some Dentists agree to the contrary.

As I have before stated, I think all kinds of materials now in use for filling teeth are sometimes practical. I have, in the "*Cosmos*" of last year, advocated the use of crystal or sponge gold quite strongly, and I still hold to the statements in the main. Two years' experience in its use has been worth something to me. I would not use it now under some circumstances, when I once would. By making a choice of operations and circumstances, I would use it, and with as good results, as with any preparation of gold extant. One condition under which I would not use it: when an unhealthy saliva exists, (thick, ropy and vitiated,) or any of the front teeth, another is against a thin crumbling enamel of a tooth, when the pulp has long remained dead under a filling. In such cases I think foil far superior. What is needed is ability to judge discreetly and wisely. I think, however, I have made more good operations in the same length of time with sponge than I have ever with foil.

I have been sorely troubled in the use of foil from the fact that there has been such a want of uniformity. The great mass of adhesive foils fail in this one point, want of softness after being annealed by the Dentists. Some lots of different makers would be all that could be desired, and another would be directly the opposite. Some that would be soft, would have no tenacity. I am told by different makers, they have had all sorts of suggestions made to them by Dentists, and I think they have tried so hard to follow them, they are often led into a muddle of ideas. It has been my conviction since participating in a discussion on adhesive gold at the Connecticut River Valley Association, held at Springfield, Mass., in Oct., 1865, that the cause of this want of softness, after annealing is simply from the presence of a foreign matter, and they are more and more confirmed, partly on account of the remarks and experience of Dr. Wetherbee, of Boston, pubilshed in the REGISTER in the December number, of 1866, and from my own experience, since that time. After reading the article by Dr. Wetherbee, I called the attention of three makers of foil to the ideas given. Mr. E. Kearsing, of Brooklyn, set himself to work and has prepared a foil that he declares to be as absolutely pure as can be, and gives me his reasons for knowing, which, to me, are so convincing, I can but acknowledge the truth of his statement. The foil he has furnished, is so far excellent, and says he can produce it every time, (the every time is what we want). I am acquainted with one other Dentist who is using some of the same and expresses himself delighted. It is as soft, tough, and adhesive after annealing, as can be desired. If he continues to give me such an article as he has, I shall use more foil than I have been accustomed to do in the last two years.

P. S.—I have of late used nearly four boxes of Lamm's gold, and from my experience with it, I can say, with a good deal of confidence, I believe it will come to be a very valuable auxiliary in filling teeth, aside from its use in connection with the fluids of the mouth.

